# Mortality in chronic kidney disease patients with COVID-19: a systematic review and meta-analysis

**DOI:** 10.1007/s11255-020-02740-3

**Published:** 2021-01-03

**Authors:** Ruyi Cai, Jinshi Zhang, Yifan Zhu, Lin Liu, Yueming Liu, Qiang He

**Affiliations:** 1 School of Medicine, Hangzhou Normal University, Hangzhou, Zhejiang 310018 People’s Republic of China; 2 Department of Nephrology, Zhejiang Provincial People’s Hospital, People’s Hospital of Hangzhou Medical College, Hangzhou, Zhejiang 310014 People’s Republic of China; 3 People’s Hospital of Hangzhou Medical College, Hangzhou, Zhejiang 310014 People’s Republic of China; 4 Chinese Medical Nephrology Key Laboratory of Zhejiang Province, Hangzhou, Zhejiang 310014 People’s Republic of China

**Keywords:** COVID-19, SARS-CoV-2, Chronic kidney disease, Mortality

## Abstract

At the beginning of 2020, the outbreak of coronavirus disease 2019 (COVID-19) led to a worldwide pandemic and mass panic. The number of infected people has been increasing exponentially since, and the mortality rate has also been concomitantly increasing. At present, no study has summarized the mortality risk of COVID-19 in patients with chronic kidney disease (CKD). Therefore, the aim of the present study was to conduct a literature review and meta-analysis to understand the frequency of mortality among CKD patients infected with COVID-19. A comprehensive systematic search was conducted on the PubMed, Embase, and Cochrane databases to find articles published until May 15, 2020. Study quality was assessed using a modified version of the Newcastle–Ottawa Scale. After careful screening based on the inclusion and exclusion criteria, 3,867,367 patients from 12 studies were included. The mortality rate was significantly higher among CKD patients with COVID-19 infection than among CKD patients without COVID-19 infection, as indicated by a pooled OR of 5.81 (95% CI 3.78–8.94, *P* < 0.00001, *I*^2^ = 30%). The patients were then stratified into ≥ 70 and < 70 years, and subgroup analysis revealed that among CKD patients with COVID-19 infection, the mortality rate was higher in the < 70 years group (OR 8.69, 95% CI 7.56–9.97, *P* < 0.0001) than in the ≥ 70 years group (*OR* 2.44, 95% CI 0.75–6.63, *P* = 0.15). Thus, COVID-19 patients with CKD have a high mortality risk and require a comprehensive multidisciplinary management strategy.

## Introduction

In December 2019, the first outbreak of coronavirus disease (COVID-19) caused by severe acute respiratory syndrome novel beta-coronavirus (SARS-CoV-2) was reported in Wuhan, China [1]. Since then, the novel coronavirus has dealt a severe blow to the global health care industry and led to mass panic all over the world [2]. As of July 1, 2020, a total of 10,596,241 confirmed cases and 514,242 deaths were reported across 212 countries (source: World Health Organization website), and the number of confirmed cases and deaths continues to rise. SARS-CoV-2 is mainly transmitted from person to person through respiratory droplets, which are usually released when an infected person coughs or sneezes. Its main clinical manifestations are fever, cough, myalgia, malaise, and diarrhea [3, 4]. The diagnosis of COVID-19 is usually based on PCR detection of SARS-CoV-2 in nasopharyngeal swabs or other specimens [5].

Patients with severe disease may present with dyspnea, a respiratory rate higher than 30 breaths per minute, blood oxygen saturation less than 93%, a PaO_2_:FiO_2_ ratio ≤ 300 mm Hg, and invasion of more than 50% of the lung fields within 24–48 h of symptom onset [6]. In patients with COVID-19, comorbidities (including cardiovascular disease, chronic kidney disease, diabetes, and other chronic diseases) are associated with a higher risk of severe complications and death [7]. In particular, chronic kidney disease (CKD) is associated with an increase in the hospitalization rate of patients with novel coronavirus-associated pneumonia, and the mortality rate seems to be 14–16 times higher than that of the general population [8]. Additionally, a meta-analysis suggested that CKD should be considered as an important risk factor for COVID-19 [9], and Cheng et al. showed that COVID-19 patients had a high prevalence of kidney disease at admission and a high in-hospital mortality rate [10]. Therefore, there is an urgent need to understand the risks in patient populations with such co-morbidities. This meta-analysis responds to this need by focusing on studies that report the risk of clinical death in patients with CKD complicated with COVID-19 and evaluating the reliability of the evidence. The purpose of this article is to explore the frequency of clinical deaths in patients with CKD complicated with COVID-19.

## Methods

### Search strategy

This meta-analysis was conducted and reported in accordance with the Preferred Reporting Items for Systematic Reviews and Meta-Analysis and Meta-Analysis of Observational Studies in Epidemiology (PRISMA) guidelines [11]. To find the relevant studies, two authors (RY Cai and JS Zhang) conducted a comprehensive search of the PubMed, EMBASE, and Cochrane databases for studies published as of May 15, 2020. The search terms used were “coronavirus,” “COVID-19,” “SARS-CoV-2,” “mortality,” “outcomes,” “chronic kidney disease,” and “chronic renal disease.” The reference lists of the eligible articles were also reviewed to search for relevant articles. The supervising authors reviewed the included studies and data.

### Inclusion and exclusion criteria

This review only includes studies on humans that were published in English and reported confirmed cases of COVID-19 in patients with CKD, with data for both survivors and non-survivors. Studies that were published in duplicate, case reports, reviews, and letters were excluded, as were studies that focused only on the mortality rate of CKD or only on COVID-19. Two authors (RY Cai and JS Zhang) independently screened the titles and abstracts of all potentially relevant studies and reviewed the full text of the articles that met the inclusion criteria.

### Data extraction and quality check

The following features were extracted from the relevant articles: first author’s name, country, source of data, sample size, and age and gender of both survivors and non-survivors. The quality of the studies was assessed using the Newcastle–Ottawa scale (NOS) [12]. Quality assessment was independently conducted by two authors (RY Cai and JS Zhang), and any disagreement was resolved via discussion.

### Statistical analysis

Our meta-analysis was performed with Review Manager 5.3. Heterogeneity was assessed by calculating the *I*^2^ index. The fixed- or random-effects model was utilized, as appropriate. Sensitivity analysis was used to evaluate the impact of each study by eliminating studies one at a time or alternating between random-effects and fixed-effects models. The findings from the meta-analysis are graphically represented by a forest plot. Publication bias was determined and presented using a funnel plot. For categorical outcomes, an odds ratio (OR) with 95% CI (for observational studies) was calculated for each study. The pooled odds ratios (ORs) of different studies and the corresponding 95% confidence intervals (CIs) were used to estimate the frequency of clinical deaths in patients with CKD complicated with COVID-19. All p-values in this study were two tailed, and statistical significance was set at ≤ 0.05.

## Results

### Characteristics of the included studies

**Fig. 1:**
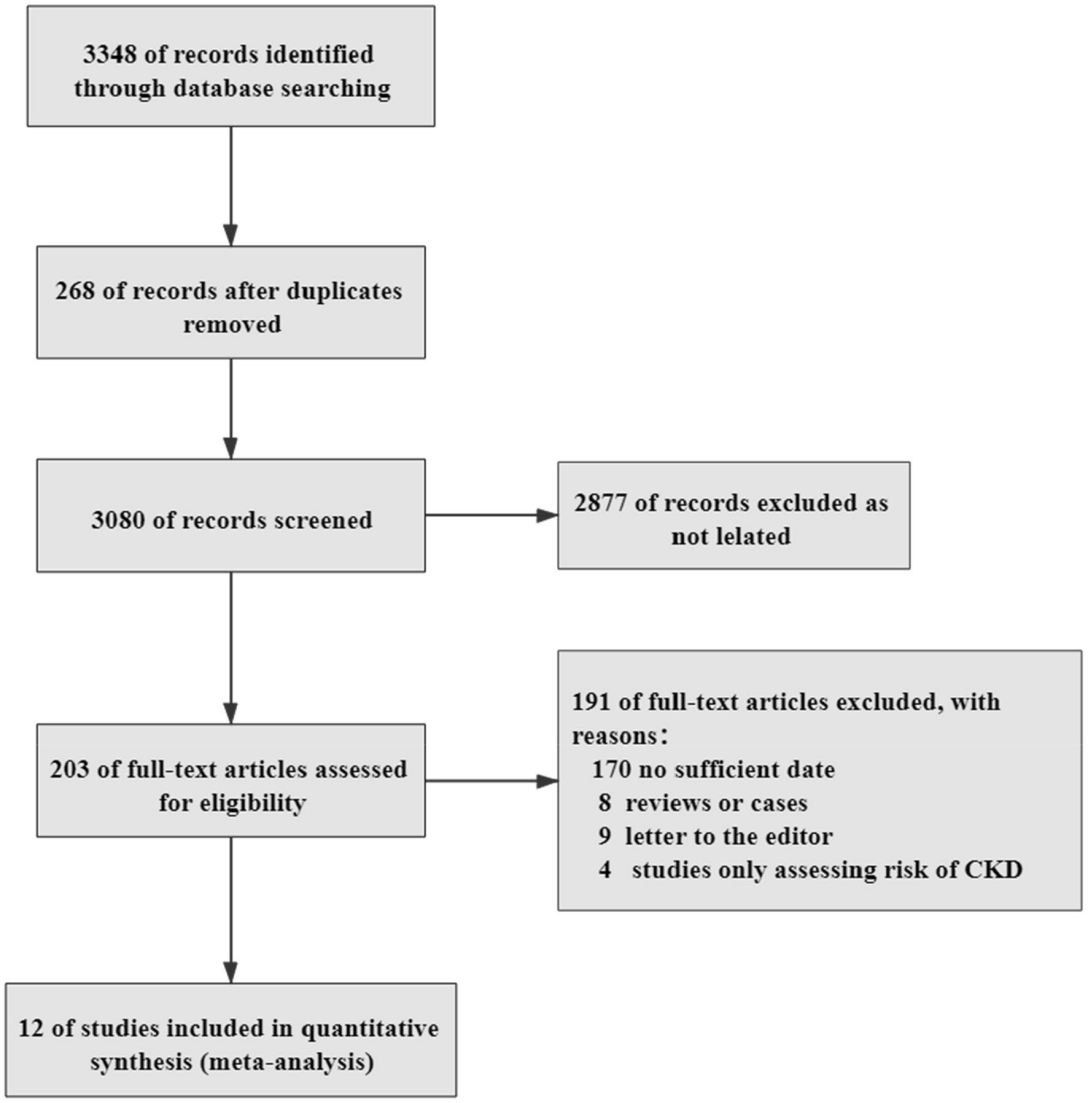
Flow chart depicting the search strategy to identify articles on the relationship between CKD and the mortality of patients with COVID-19

A total of 3348 articles were selected from the databases, but 268 were excluded because of duplication. The titles and abstracts of the articles were screened, and 2877 were excluded because they did not meet the inclusion criteria. After the full text of the articles was read, 191 articles with inconsistent data were deleted. Finally, 12 articles that met all the inclusion criteria were included [13–24]. The selection process is depicted in Fig. 1, and the common baseline characteristics of the patients are shown in Table 1. The NOS score of the included studies was 7–8, and the quality of the articles was evaluated as high (Table 2).

**Table 1:** Baseline characteristics of studies included in the systematic review

Author	Source of data	Country	Dead (*n*, %)	Mean/median age (years)	Female (%)
Age ≥ 70 years					
Chen et al. [22]	Zhongnan Hospital of Wuhan University	China	19, 34.55%	74	38.18
Wang et al. [15]	Renmin Hospital of Wuhan University	China	65, 19.17%	71	51.03
Age < 70 years					
Banerjee et al. [24]	Hospitals in UK	England	38,847, 1.01%	48	50.70
Cao et al. [23]	Wuhan University Zhongnan Hospital	China	17, 16.67%	54	48.04
Chen et al. [21]	Tongji Hospital	China	113, 41.54%	62	37.87
Nikpouraghdam et al. [20]	Baqiyatallah Hospital	China	239, 8.06%	56	34.04
Ruan et al. [19]	Tongji Hospital	China	68, 45.33%	58.5	32
Shi et al. [17]	Renmin Hospital of Wuhan University	China	62, 9.24%	63	52.01
Shi et al. [18]	Renmin Hospital of Wuhan Zhongnan Hospital	China	47, 15.36%	64.5	50.98
Wang et al. [16]	Zhongnan Hospital Xishui Hospital	China	19, 17.76%	51	46.73
Yan et al. [14]	Tongji Hospital	China	108, 54.55%	64	39.90
Zhou et al. [13]	Jinyintan Hospital Wuhan Pulmonary Hospital	China	54, 28.27%	56	37.70

**Table 2:** Newcastle–Ottawa score of the included studies

Study ID	Exposed cohort representative	Non-exposed cohort selected from same source	Exposure ascertained	Outcome of study was not present at start of the study	Comparability	Adequate assessment	Follow-up was long enough	Adequate follow-up	Quality score
Chen et al. [22]	Yes	Yes	Yes	Yes	0	Yes	Yes	Yes	7
Wang et al. [15]	Yes	Yes	Yes	Yes	0	Yes	Yes	Yes	7
Banerjee et al. [24]	Yes	Yes	Yes	Yes	0	Yes	Yes	Yes	7
Cao et al. [23]	Yes	Yes	Yes	Yes	0	Yes	Yes	Yes	7
Chen et al. [21]	Yes	Yes	Yes	Yes	1	Yes	Yes	Yes	8
Nikpouraghdam et al. [20]	Yes	Yes	Yes	Yes	0	Yes	Yes	Yes	7
Ruan et al. [19]	Yes	Yes	Yes	Yes	0	Yes	Yes	Yes	7
Shi et al. [17]	Yes	Yes	Yes	Yes	0	Yes	Yes	Yes	7
Shi et al. [18]	Yes	Yes	Yes	Yes	0	Yes	Yes	Yes	7
Wang et al. [16]	Yes	Yes	Yes	Yes	0	Yes	Yes	Yes	7
Yan et al. [14]	Yes	Yes	Yes	Yes	0	Yes	Yes	Yes	7
Zhou et al. 13	Yes	Yes	Yes	Yes	0	Yes	Yes	Yes	7

### Pooled analysis

**Fig. 2:**
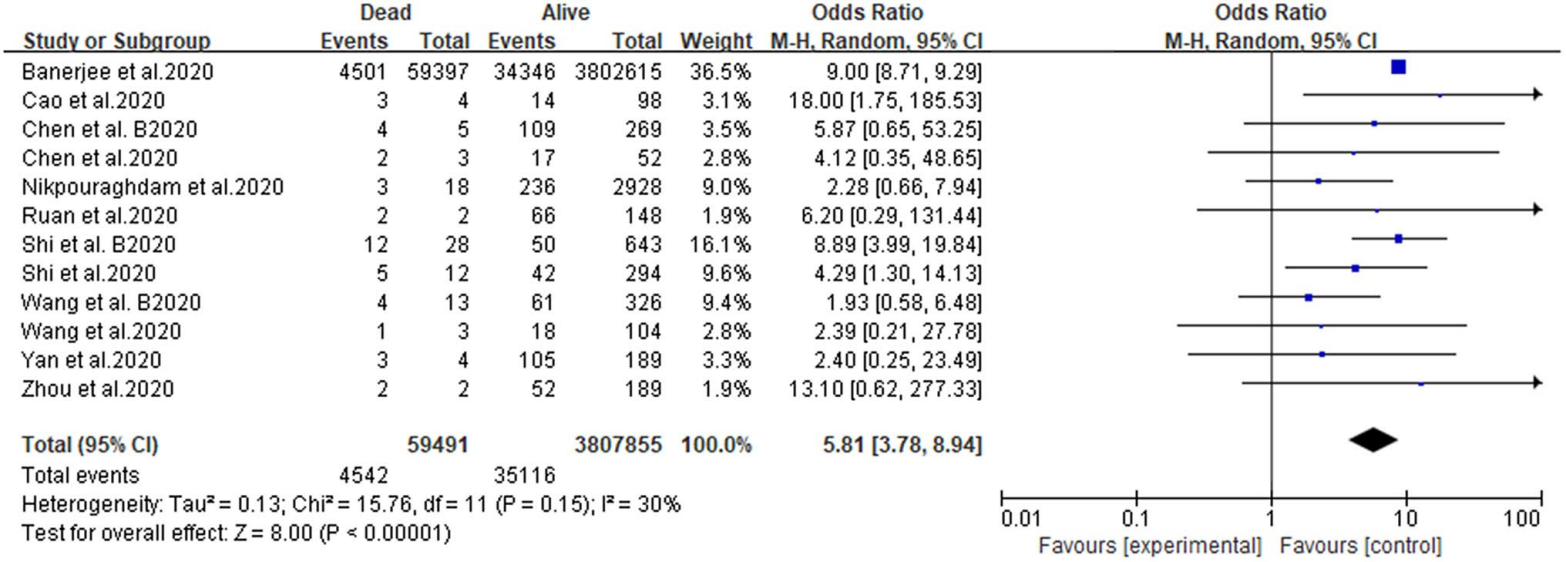
Forest plot depicting the relationship between CKD and the mortality of patients with COVID-19

The forest plot of mortality in patients with CKD infected with COVID-19 is shown in Fig. 2. REM analysis showed that CKD patients with COVID-19 had a higher mortality rate (pooled OR 5.81, 95% CI 3.78–8.94, *P* < 0.00001, *I*^2^ = 30%). As shown in Fig. 2, although most of the samples were from the UK, this did not create a bias in the study results.

### Subgroup analysis and sensitivity analysis

**Fig. 3:**
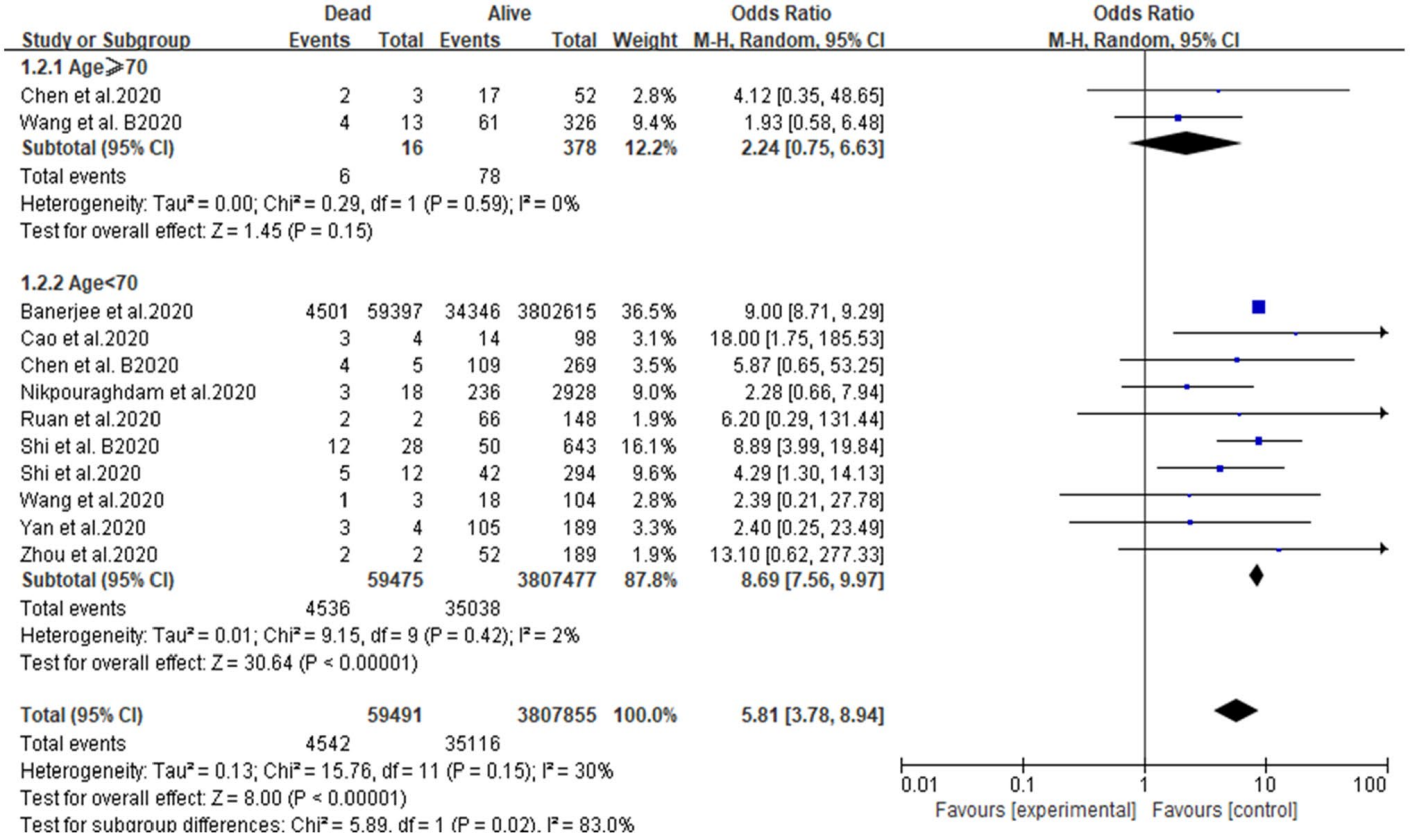
Forest plot depicting the relationship between CKD and the mortality of COVID-19 patients after subgroup analysis according to age

**Fig. 4:**
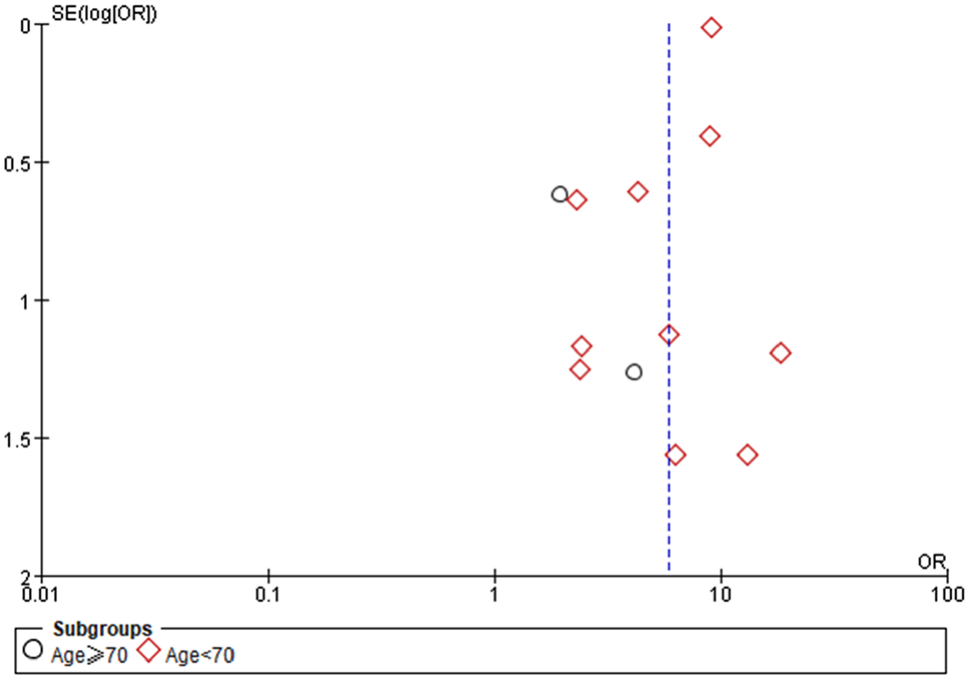
Funnel plot depicting publication bias

Patients were divided into two subgroups according to their age: ≥ 70 years and < 70 years. The results of the two groups were significantly different (≥ 70 years: OR 2.44, 95% CI 0.75–6.63, *P* = 0.15; < 70 years: OR 8.69, 95% CI 7.56–9.97, *P* < 0.0001) (Fig. 3). When all the studies were analyzed, low heterogeneity was observed (*I*^2^ = 30%), but after the studies of Chen and Wang were excluded, the heterogeneity decreased significantly (*I*^2^ = 2%). Therefore, Chen and Wang’s study may be responsible for the heterogeneity of this meta-analysis. There was no evidence of a publication bias, according to the findings of funnel plot analysis (Fig. 4).

## Discussion

This study presents a literature review and meta-analysis of the information reported so far on the mortality rate of CKD patients with COVID-19. According to the literature review in the current study, no meta-analysis has analyzed the mortality of CKD patients with COVID-19. Nonetheless, based on the data of the relevant studies extracted from the literature, our research found that COVID-19 infection was closely associated with mortality in CKD patients. Although cardiovascular disease is the leading cause of death in CKD, the death rate associated with pulmonary infection in CKD patients is also high [25, 26]. Our findings confirm this, as the CKD patients with COVID-19 infection had a higher mortality rate than those without COVID-19 infection (pooled OR 5.81). This is probably because in CKD patients, the levels of pro-inflammatory cytokines are increased, and this leads to an increase in oxidative stress that eventually produces an inflammatory immune response. The resulting immune system damage may increase susceptibility to bacterial and viral infections, and this might be the main reason for the increased risk of pulmonary inflammation [27]. When the patients in the current meta-analysis were stratified into those older than 70 years and those younger than 70 years (≥ 70 years: OR 2.44, 95% CI 0.75–6.63, *P* = 0.15; < 70 years: OR 8.69, 95% CI 7.56–9.97, *P* < 0.0001), we found that among CKD patients complicated with COVID-19, those younger than 70 years had a higher mortality rate than those who were 70 years or older. This is probably because elderly patients are more likely to have other complications (such as hypertension, diabetes, coronary heart disease, and COPD). These comorbidities will increase the risk of death associated with COVID-19 in the elderly, so the specific condition associated with the increased risk of death in CKD patients is not clear. Moreover, the high mortality rate in elderly patients is probably related to the higher incidence of these comorbidities, rather than CKD [28]. However, misdiagnosis or delayed diagnosis of COVID-19 is common in elderly patients with CKD, on account of low immune response, mild clinical symptoms, and atypical CT imaging findings of the chest [29]. This may explain why an increase in mortality is not found in elderly CKD patients with COVID-19 infection. As CKD can be prevented and treated to a large extent, it deserves more attention in health policy decision-making globally. Therefore, it is necessary to establish a comprehensive disease management strategy that includes lifestyle, nutrition, medications, and other methods to reduce the mortality rate of CKD patients.

Our study has several limitations. One of the main limitations is that there were no data about the disease stage of the CKD patients, the disease course, and the drugs that were administered. Second, the data were not adjusted for confounding factors, such as gender, race, and BMI, which may have affected the results. Third, CKD patients with COVID-19 may have other chronic diseases (such as hypertension, diabetes, COPD, and cardiovascular and cerebrovascular diseases) that may have affected the results, but the related data were not considered in the analysis. Fourth, the age stratification carried out was not comprehensive enough.

## Conclusion

The results of the present meta-analysis on the limited published data indicate that regardless of age, CKD patients with COVID-19 infection have a high mortality risk. Thus, CKD patients infected with SARS-CoV-2 must be carefully monitored and managed to lower the risk of death.

## Data Availability

All data needed to evaluate the conclusions in the paper are present in the paper.
